# Prevalence, predictors, and prognostic implications of residual impairment of functional capacity after transcatheter aortic valve implantation

**DOI:** 10.1007/s00392-017-1119-9

**Published:** 2017-04-25

**Authors:** Mohammad Abdelghani, Rafael Cavalcante, Yosuke Miyazaki, Robbert J. de Winter, Rogério Sarmento-Leite, José A. Mangione, Alexandre Abizaid, Pedro A. Lemos, Patrick W. Serruys, Fabio S. de Brito

**Affiliations:** 1 Department of Cardiology, The Academic Medical Center, University of Amsterdam, Room TKs0-248, Meibergdreef 9, 1100 DD Amsterdam, The Netherlands; 2 Thoraxcenter, Erasmus Medical Center, Rotterdam, The Netherlands; 3 Instituto de Cardiologia do Rio Grande do Sul/Fundação Universitária de Cardiologia and Universidade Federal de Ciências da Saúde de Porto Alegre, Porto Alegre, RS Brazil; 4 Hospital Beneficência Portuguesa de São Paulo, São Paulo, Brazil; 5 Instituto Dante Pazzanese de Cardiologia, São Paulo, Brazil; 6 Hospital Israelita Albert Einstein, São Paulo, Brazil; 7 The Heart Institute (InCor), University of São Paulo Medical School, São Paulo, Brazil; 8 International Centre for Circulatory Health, NHLI, Imperial College London, London, UK

**Keywords:** Aortic valve stenosis, TAVI, TAVR, Functional capacity, Quality of life

## Abstract

**Background:**

Patients with degenerative aortic stenosis (AS) referred for transcatheter aortic valve implantation (TAVI) typically have advanced cardiac and vascular adverse remodeling and multiple comorbidities and, therefore, might not recover a normal functional capacity after valve replacement. We sought to investigate the prevalence, the predictors, and the prognostic impact of residual impairment of functional capacity after TAVI.

**Methods and results:**

Out of 790 patients undergoing TAVI with impaired functional capacity (NYHA II–IV) at baseline, NYHA functional class improved in 592 (86.5%) and remained unchanged/worsened in 92 (13.5%) at follow-up [median (IQR): 419 (208–807) days] after TAVI. Normal functional capacity (NYHA I) was recovered in 65.5% (*n* = 448) of patients, while the rest had variable degrees of residual impairment. On multivariable regression analysis, atrial fibrillation [odds ratio-OR, 2.08 (1.21–3.58), *p* = 0.008], low-flow–low-gradient AS [OR, 1.97 (1.09–3.57), *p* = 0.026], chronic obstructive pulmonary disease [OR, 1.92 (1.19–3.12), *p* = 0.008], and lower hemoglobin at baseline [OR, 1.11 (1.01–1.21) for each g% decrement, *p* = 0.036] were independently associated with residual impairment of functional capacity. All-cause and cardiac mortality were significantly higher in those with residual impairment of functional capacity than in those in NYHA I class [hazard ratio-HR: 2.37 (95% CI: 1.51–3.72), *p* < 0.001 and 2.16 (95% CI: 1.08–4.35), *p* = 0.030, respectively]. Even mild residual functional impairment (NYHA II) was associated with a higher all-cause [HR: 2.02 (95% CI: 1.10–3.72), *p* = 0.023] and cardiac [HR: 2.08 (95% CI: 1.42–3.07), *p* < 0.001] mortality.

**Conclusion:**

Residual impairment of functional capacity is common after TAVI and is independently associated with increased mortality. Predictors of residual impairment of functional status are predominantly patient-rather than procedure-related.

**Electronic supplementary material:**

The online version of this article (doi:10.1007/s00392-017-1119-9) contains supplementary material, which is available to authorized users.

## Introduction

Patients with severe aortic stenosis (AS) typically have symptoms of heart failure and impaired quality of life and are subject to increased mortality and escalation of symptoms, once they have developed [1].

Patients with degenerative AS referred for transcatheter aortic valve implantation (TAVI) typically have advanced cardiac [2] and vascular [3] adverse remodeling that may not be completely reversible after valve replacement [2, 4, 5]. In addition, non-cardiovascular comorbidities are common in those patients [2]. Therefore, although TAVI can modify the dismal natural history of severe AS, restoration of a normal functional capacity may be less likely to occur. Although the major TAVI pivotal trials reported similar functional improvement after TAVI vs. surgical aortic valve replacement (SAVR) [6–8], a recent meta-analysis of randomized trials of patients at low and intermediate risks of perioperative mortality showed that transfemoral TAVI was associated with reduced mortality but increased incidence of heart failure within 2 years as compared to SAVR [9].

Consequently, TAVI might remain a futile treatment in patients who are more concerned with their functional status than with the risk of death. Given the fact that all TAVI candidates expect an improvement of their quality of life after the procedure [10] and that some patients are more concerned with their functional status than with the risk of death, it is desirable to know the likelihood, the predictors, and the prognostic implications of failure to recover a normal functional capacity after the procedure. We specifically sought to identify whether residual impairment of functional capacity is linked to more advanced cardiopathy and severer symptoms at baseline, to non-cardiac comorbidities, or to procedural failure.

## Methods

The study included consecutive patients enrolled by 22 centers in the Brazilian TAVI registry from January 2008 to January 2015. Patients were considered eligible for inclusion if they had severe symptomatic AS (of the native valve or of a degenerated bioprosthetic surgical valve) and were considered by the heart team as inoperable or at high surgical risk. Operative mortality risk was estimated using the logistic European System for Cardiac Operative Risk Evaluation (EuroSCORE) and the Society of Thoracic Surgeons Predicted Risk of Mortality (STS-PROM) risk scores. List of participating centers, details of TAVI-procedure technical aspects, and adjudication of adverse events have been previously described elsewhere [11]. The study protocol complies with the Declaration of Helsinki and was approved by the ethics committee at each of the participating centers and all patients provided written informed consent. A web-based case report form and remote electronic data monitoring were utilized with an on-site source document validation performed in a random sample (one-fifth of all cases). An independent committee (including a neurologist) adjudicated all events. All endpoints are reported according to the Valve Academic Research Consortium-2 (VARC-2) criteria [12]. Device failure was defined as residual transaortic mean pressure gradient ≥20 mmHg, greater than mild aortic regurgitation, and/or failure to correctly position a single device into the proper anatomical location [12].

Symptoms related to AS included: impaired functional capacity, angina, syncope, and/or pre-syncope [13]. According to the New York Heart Association (NYHA) functional classification, patients in NYHA class I have a normal functional capacity and are free from symptoms attributable to heart disease, and those in NYHA classes II, III, and IV have mild, moderate, and severe impairments of functional capacity due to symptoms attributable to heart disease, respectively [14].

Transfemoral vascular access was the default approach with the use of alternative approaches (transubclavian, direct transaortic, and transcarotid) only when the transfemoral access was not possible. The decision to choose between sedative or general anesthesia was left to the discretion of the operators. All patients underwent transthoracic echocardiographic (TTE) study at baseline and were scheduled for TTE during the same admission for the index procedure (pre-discharge TTE) and for follow-up at 6 and 12 months and annually thereafter.

### Statistical analysis

Quantitative variables are summarized as mean ± SD or median (interquartile range-IQR) and are compared by Student *t* test or Mann-Whitney U test, while categorical variables are summarized as frequencies and proportions and are compared with the use of the Chi-square test.

Uni- and multivariable logistic regression analyses were used to identify the factors potentially associated with residual impairment of functional capacity. Factors with a *p* value <0.10 in univariable analysis were included in a stepwise multivariable logistic model.

Cumulative survival curves for patients with and without residual impairment of functional capacity were constructed using the Kaplan–Meier method and compared with the log-rank test and Cox-proportional hazards model.

All analyses were performed with SPSS 23 (IBM, Armonk, NY, USA). All probability values were two-tailed, and a *p* value <0.05 was considered significant.

## Results

A total of 819 consecutive patients with severe symptomatic AS were included (mean age 81.5 ± 7.3 years; 49% males). Patients were at high surgical risk (EuroSCORE, 20.5 ± 14.7; STS score, 10.3 ± 7.8) with a high burden of comorbidities (chronic kidney disease, 77%; coronary artery disease, 59%; and chronic obstructive pulmonary disease, 19%). TAVI was preformed predominantly under general anesthesia (91%) through a transfemoral access (93%), and involved implanting a CoreValve (73%) or a Sapien-XT (24%) device in the majority of cases.

Before TAVI, 790 patients (96%) had impaired functional capacity [NYHA II in 124 patients (15%), NYHA III in 436 patients (53%), and NYHA IV in 230 patients (28%)]. Among patients with impaired functional capacity (NYHA ≥ II) at baseline, 684 were alive beyond 30 days post-procedure and available for clinical follow-up [up to 2268 days, median (IQR): 419 (208–807) days]. Out of those, NYHA functional class improved in 592 (86.5%) and remained unchanged/worsened in 92 (13.5%) (Supplementary Figure). Ultimately, 65.5% of patients (*n* = 448) had recovered a normal functional capacity (NYHA I), while the rest had variable degrees of residual impairment. The latter was mild (NYHA II) in 26.5% (*n* = 183) and moderate–severe (NYHA III or IV) in 8% (*n* = 53).

### Characteristics of patients with residual impairment of functional capacity

The baseline, periprocedural, and follow-up characteristics in the patients stratified according to the functional status at follow-up are summarized in a Supplementary Table. All relevant baseline and periprocedural factors were tested for association with residual impairment of functional capacity after TAVI. Table 1 summarizes the univariable and multivariable predictors.

**Table 1:** Univariable and multivariable predictors of residual impairment of functional capacity among survivors beyond 30 days after TAVI

	Univariate analysis				Multivariate analysis			
	OR	Lower 95% CI for OR	Upper 95% CI for OR	*p*	OR	Lower 95% CI for OR	Upper 95% CI for OR	*p*
Body mass index (kg/m^2^) at baseline	0.968	0.935	1.002	0.065	0.958	0.916	1.001	0.054
EuroSCORE at baseline	1.013	1.002	1.024	0.019	1.002	0.986	1.018	0.812
NYHA functional class at baseline	1.269	1.026	1.569	0.028	0.981	0.744	1.293	0.893
Pulmonary hypertension at baseline	1.436	0.992	2.078	0.055	0.853	0.519	1.401	0.531
Atrial fibrillation/flutter at baseline	2.019	1.280	3.186	0.003	**2.084**	**1.213**	**3.582**	**0.008**
LV posterior wall thickness (mm) at baseline	0.925	0.852	1.003	0.061	0.950	0.865	1.044	0.288
LV ejection fraction (%) at baseline	0.991	0.980	1.001	0.072	1.000	0.983	1.017	0.984
Transaortic valve mean PG (mmHg) at baseline	0.989	0.979	0.999	0.035	1.002	0.988	1.016	0.761
Low-flow–low-gradient AS at baseline	2.285	1.390	3.757	0.001	**1.968**	**1.086**	**3.568**	**0.026**
Hemoglobin (g%) at baseline	1.112	1.029	1.189	0.010	**1.114** ^a^	**1.008**	**1.209**	**0.036**
Creatinine clearance (ml/min) at baseline	0.990	0.982	0.997	0.008	0.995	0.985	1.006	0.387
Chronic obstructive pulmonary disease at baseline	1.736	1.170	2.575	0.006	**1.922**	**1.186**	**3.115**	**0.008**
Device failure^b^	1.730	1.041	2.875	0.034	1.304	0.678	2.505	0.426

Bold values indicate the covriates that are signficantly associated with residual impairment of functional capacity in multivariable regression analysis

*AS* aortic stenosis, *CI* confidence interval, *LV* left ventricle, *NYHA* New York Heart association, *OR* odds ratio

^a^Odds ratio calculated per 1 g% decrement

^b^Defined as residual transaortic mean pressure gradient ≥20 mmHg, greater than mild aortic regurgitation, and/or failure to correctly position a single device into the proper anatomical location

On multivariable logistic regression analysis, atrial fibrillation/flutter [odds ratio-OR, 2.08 (1.21–3.58), *p* = 0.008], low-flow–low-gradient AS [OR, 1.97 (1.09–3.57), *p* = 0.026], chronic obstructive pulmonary disease [OR, 1.92 (1.19–3.12), *p* = 0.008], and lower hemoglobin [OR, 1.11 (1.01–1.21) for each g% decrement, *p* = 0.036] were independently associated with residual impairment of functional capacity after TAVI. Although device failure (mainly driven by a higher trans-prosthetic valve pressure gradient-Supplementary Table) was associated with residual impairment of functional capacity in univariable analysis [OR, 1.73 (1.04–2.88), *p* = 0.034], it was not an independent predictor in the multivariable analysis.

### Cardiac remodeling in patients with recovered vs. impaired functional capacity after TAVI

**Fig. 1:**
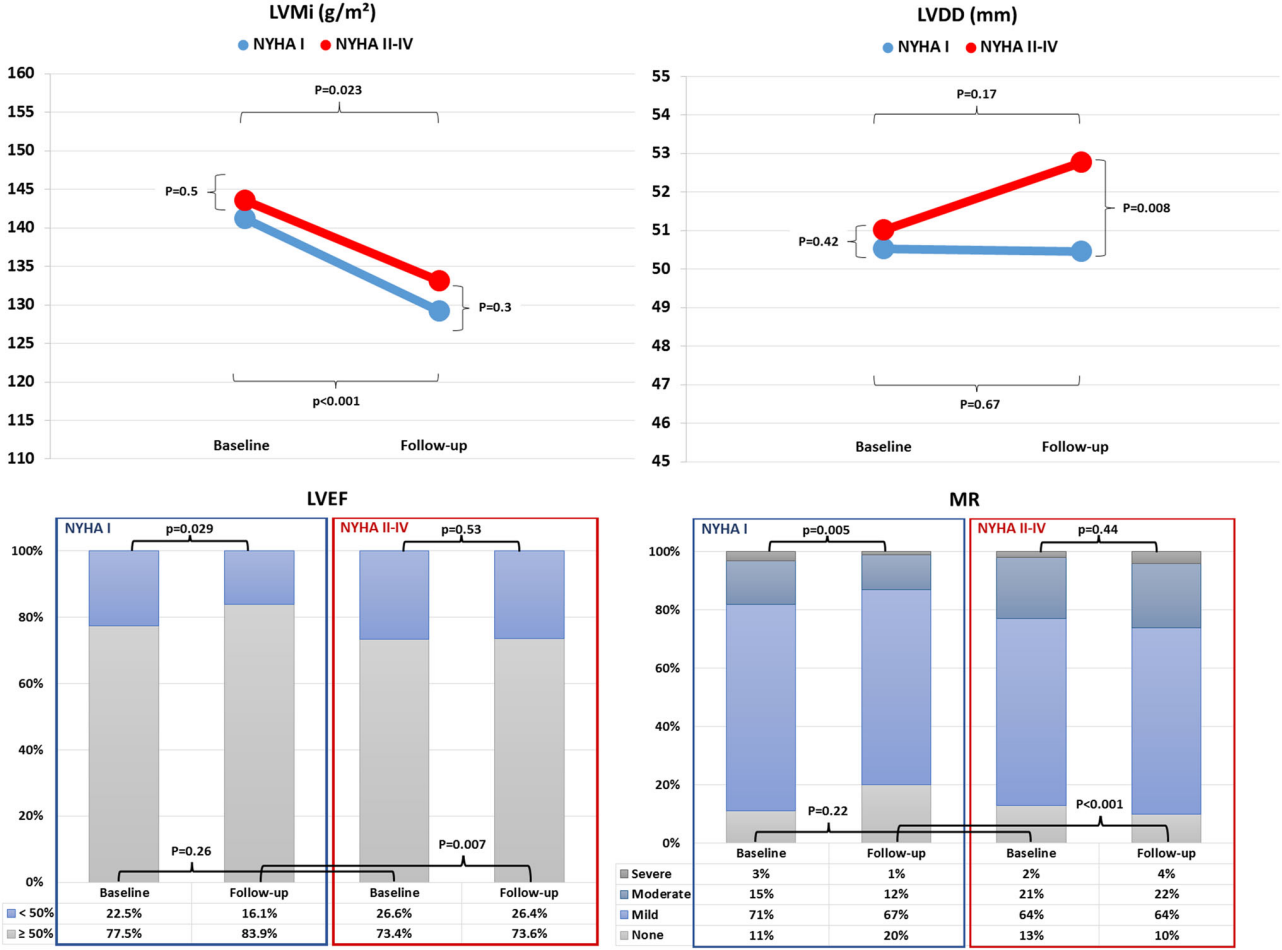
Change from baseline to latest follow-up in left ventricular mass index (LVMi), LV diastolic diameter (LVDD), LV ejection fraction (LVEF), and mitral regurgitation (MR) in patients with recovered vs. impaired functional capacity after TAVI

Echocardiographic follow-up was available in 532 patients and was performed at a median interval of 366 (161–736) days after TAVI. As shown in Fig. 1, apart from left ventricular mass index (LVMi) which improved significantly in both groups with no between-group difference at follow-up, reverse cardiac remodeling was less efficient in patients with residual impairment of functional capacity.

LV diastolic diameter (LVDD), although similar at baseline, was significantly larger in those with residual impairment of functional capacity at follow-up (52.8 ± 9.7 vs. 50.4 ± 8.8, *p* = 0.008). Impaired LV ejection fraction (LVEF <50%) was significantly less at follow-up as compared to baseline only in those with NYHA I symptoms. Accordingly, although not significantly different at baseline, the prevalence of impaired LVEF at latest follow-up was significantly higher in those with residual impairment of functional capacity (26.4 vs. 16.2%, *p* = 0.007). Mitral regurgitation (MR) severity was similar in both groups at baseline and significantly improved at latest follow-up in those with NYHA I symptoms (moderate–severe MR, 13% at follow-up vs. 18% at baseline, *p* = 0.005) but not in those with residual impairment of functional capacity (moderate–severe MR, 26% at follow-up vs. 23% at baseline, *p* = 0.44). Consequently, MR severity at follow-up was significantly higher in those with residual impairment of functional capacity than in those with NYHA I symptoms (moderate–severe MR, 26% vs. 13%, *p* < 0.001).

### Mortality in TAVI patients stratified according to the functional status at follow-up

**Fig. 2:**
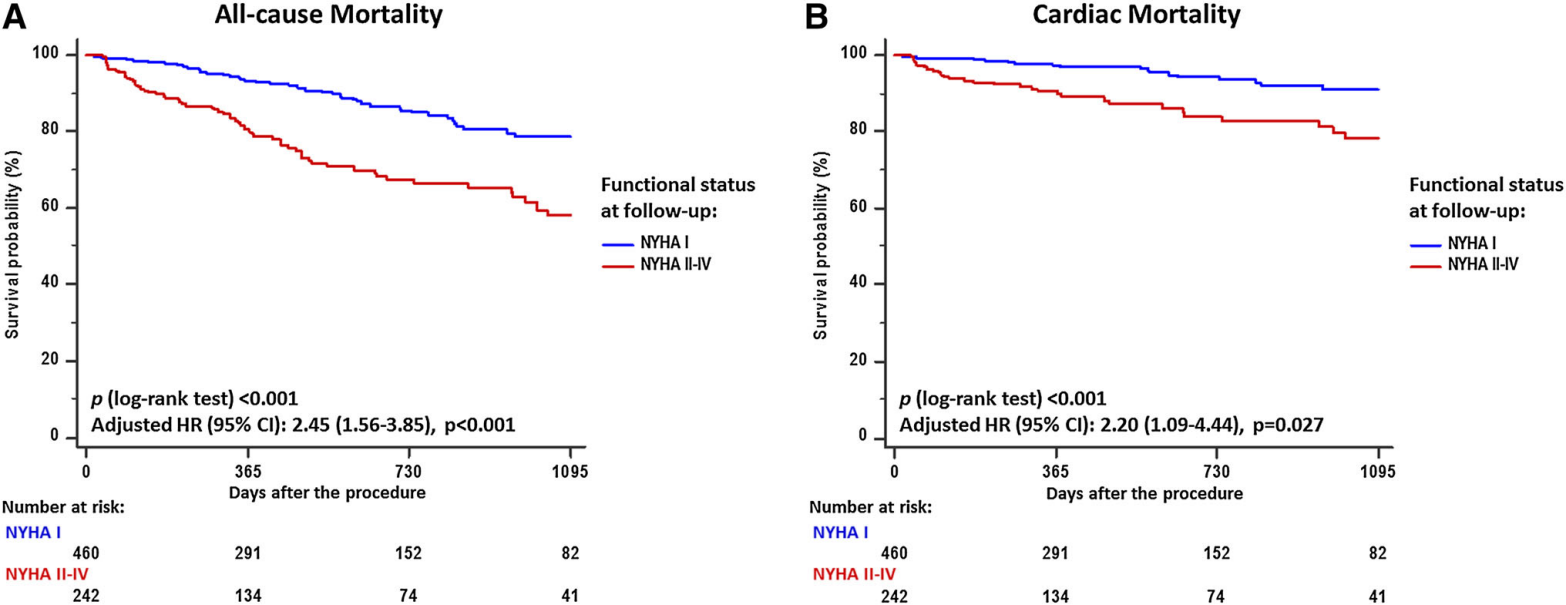
Kaplan–Meier survival curves for all-cause (**a**) and cardiac (**b**) death according to the functional status (normal vs. impaired) at follow-up after TAVI. *CI* confidence interval, *HR* hazard ratio

During the entire follow-up period, all-cause mortality was significantly higher in those with residual impairment of functional capacity than in those who recovered a normal functional status (32.6 vs. 12.7%, log-rank *p* < 0.001) (Fig. 2a). Similarly, cardiac mortality was significantly higher in those with impaired functional capacity (14.4 vs. 5.4%, *p* < 0.001) (Fig. 2b). After adjustment for the aforementioned LV remodeling markers (LVEF, LVDD, and MR at latest follow-up), the association between residual impairment of functional capacity and all-cause mortality [hazard ratio-HR: 2.37 (95% CI: 1.51–3.72), *p* < 0.001] and cardiac mortality [HR: 2.16 (95% CI: 1.08–4.35), *p* = 0.030] remained significant.

**Fig. 3:**
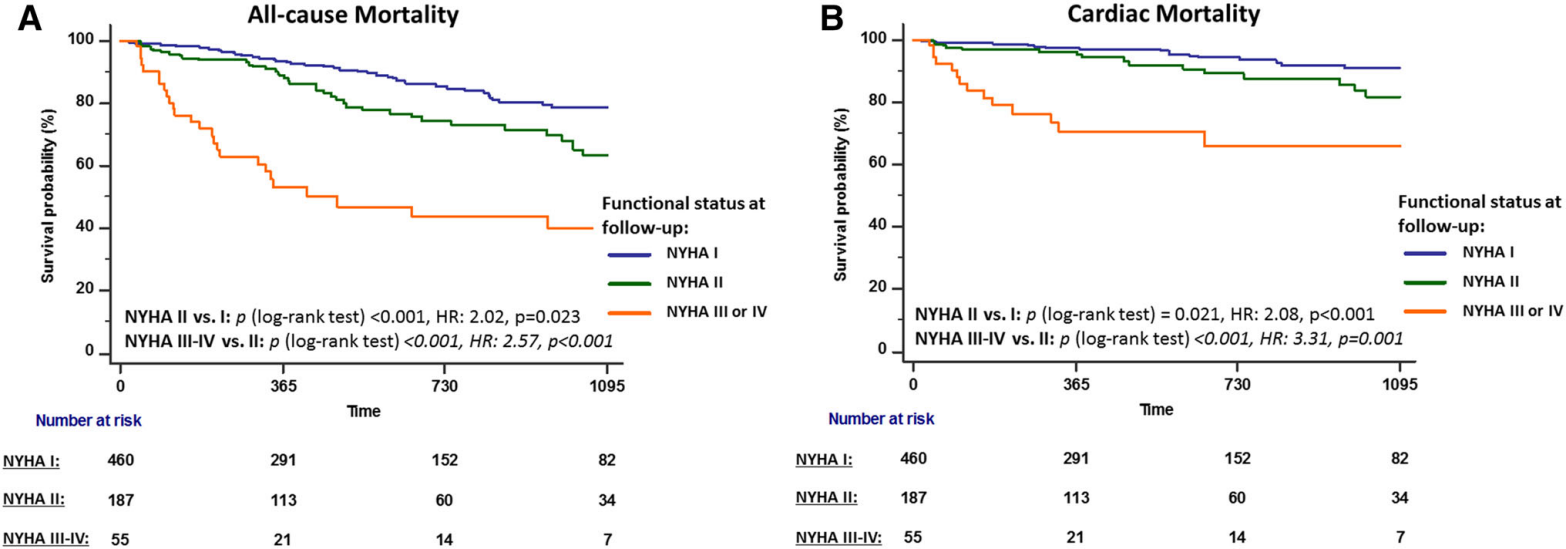
Kaplan–Meier survival curves for all-cause (**a**) and cardiac (**b**) death according to the functional status (normal vs. mildly-impaired vs. moderate–severely impaired) at follow-up after TAVI. *CI* confidence interval, *HR* hazard ratio

To explore whether residual mild impairment of functional capacity (NYHA II) after TAVI can be detrimental, survival analysis was repeated after dividing the patients into three groups; normal (NYHA I), mildly-impaired (NYHA II), and moderate–severely impaired functional capacity (NYHA III or IV). All-cause mortality was higher in those with mild impairment (26.4%) than in those who recovered a normal functional capacity [12.7%, log-rank *p* < 0.001, HR: 2.02 (95% CI: 1.10–3.72), *p* = 0.023]. Cardiac mortality was also higher in those with mild impairment (10.4%) than in those who recovered a normal functional capacity [5.4%, log-rank *p* = 0.021, HR: 2.08 (95% CI: 1.42–3.07), *p* < 0.001]. In patients who had a residual moderate–severe functional impairment, mortality was very high (53.7%, with 27.8% being cardiac) and was significantly higher than those with mild impairment [all-cause mortality: log-rank *p* < 0.001, HR: 2.57 (95% CI: 1.60–4.11), *p* < 0.001; cardiac death: log-rank *p* < 0.001, HR: 3.31 (95% CI: 1.68–6.53), *p* = 0.001]. Survival curves for the three groups are displayed in Fig. 3.

## Discussion

In the present study, we found that the majority of AS patients recover a normal functional status after TAVI despite the extensive comorbidities and the advanced cardiopathy they have at baseline. Eighty-seven percent of patients gained some improvement of their functional status (of at least one NYHA class) and moderate–severe impairment of functional capacity was reduced from 81% before to 8% after TAVI. Those who remained symptomatic (NYHA II or more), not only had their functional capacity impaired, but also had an increased risk of all-cause and cardiac death. The increased risk of mortality was not confined to those with moderate–severe residual impairment of functional capacity but also involved those with mild residual impairment, emphasizing that restoration of a normal functional capacity should be the clinical objective in TAVI patients. These results also suggest that this simple tool (NYHA functional classification) which has long been one of the main criteria for deciding the timing of intervention for AS [13, 15] can also be used as a prognostic marker after valve replacement.

Although TAVI penetration and indications are expanding, there is also an increasing awareness of that some patients offered this expensive therapy fail to derive a functional, morbidity, and/or mortality benefit from it [16]. Futility of TAVI, which can be defined as the lack of survival/functional improvement in the short term (6 months to 1 year) [16], is still an underestimated problem. The present study provides that a set of baseline characteristics of patients who, in spite of TAVI, frequently fail to recover a normal functional status and to reverse the adverse cardiac remodeling and who also have an increased mortality, so that they can be identified and appropriately-counseled up-front of the procedure.

### Assessment of TAVI outcome: patients’ vs. physicians’ perspectives

The dismal prognosis of symptomatic severe AS if managed conservatively drew the interest to developing prognosis-modifying strategies. TAVI emerged as a prognosis-modifying intervention with an un-equivocal mortality benefit compared to conservative management in patients who cannot undergo surgery [17] and compared to surgical management in high-risk [8] and intermediate-risk [6, 18] patients. However, physicians’ and patients’ appraisal of risks and benefits may differ [19], and symptomatic relief is, for some patients, a priority. In a study by Hussain et al., the majority of patients undergoing SAVR for severe AS were willing to accept considerably higher risk of perioperative death than what is considered by physicians/guidelines as “acceptable” [20]. Patients who had more severe symptoms and lower quality of life as well as those with pulmonary disease, impaired LVEF, or lower transaortic pressure gradient were more likely to accept a high/prohibitive risk of perioperative death if a normal health is likely to be restored after valve replacement [20]. These results emphasize the importance of symptomatic improvement (vs. mere survival) among the priorities of AS patients especially those with more severe symptoms.

### How much of the response to TAVI is predictable?

In the present study, two cardiac (atrial fibrillation and low-flow–low-gradient AS) and two non-cardiac (COPD and anemia) baseline clinical conditions were identified as independent predictors of impaired functional status after TAVI. The benefit of identifying these markers during the decision-making process prior to TAVI is twofold: (1) to predict the functional outcome and counsel the patient in light of the lower probability of restoring a normal functional capacity and (2) to stimulate correction of these conditions when possible knowing that failure to control these conditions will impair the functional gains from TAVI. Although not among the independent predictors, two markers of cardiac hemodynamics (transaortic valve PG and brain natriuretic peptide) seem to have an added value to the LVEF in predicting functional status after TAVI (Supplementary Table). This finding is in line with previous studies that concluded that the indices of LV mechanics other than the volumetric LVEF (e.g., longitudinal strain [21]) as well as markers of elevated LV pressure (e.g., brain natriuretic peptide [22]) are crucial in predicting the functional status in patients with severe AS. In fact, a “low-flow” status leading to a low-gradient severe AS reflects the combination of a small LV cavity, a severe diastolic dysfunction, and an impaired longitudinal contractility. The combination of both low transvalvular gradient and low ejection fraction portends significantly worse outcomes [23, 24]. These data together might explain why the mere reduction of LVEF at baseline was not an independent predictor of the functional outcomes after TAVI, while the combination of reduced LVEF and relatively low transaortic valve PG was.

Many attempts have been made to improve the predictability of TAVI outcomes, including the development of specific TAVI outcome-prediction scores. Although the inclusion of frailty and functional parameters into the predictive models has improved their performance as compared to surgical risk models, the accuracy of those models remain modest [16]. The complexity of the cardiovascular morbidity in patients with severe degenerative AS probably plays an important role in this suboptimal performance of predictive models.

Reduced arterial compliance is an important contributor to the increased afterload and to the adverse cardiac remodeling in AS patients [25]. This arterial component of the AS disease complex is likely even more pronounced in TAVI candidates, who are typically older with multiple risk factors for atherosclerosis, than SAVR candidates. In AS patients referred for valve replacement, a higher arterial stiffness correlates with less LV mass regression and with more adverse cardiac events after SAVR [26] and TAVI [27]. Yotti et al. [4] studied arterial function before and after TAVI and reported an increase in arterial load after the procedure resulting in a residual elevation of LV pressure in 70% of patients. Moreover, myocardial response to AS involves variable degrees of myocardial fibrosis [28], the extent of which correlates with NYHA functional class at baseline [29], and predicts the improvement in NYHA class after valve replacement [29]. Arterial stiffness and myocardial fibrosis are two examples of important contributors to the impaired cardiac performance in AS patients that might attenuate the benefit from TAVI. Therefore, the classic screening of patient’s symptoms, comorbidities and valvular/myocardial function might not reflect the complete spectrum of the actual patient morbidity.

### Limitations

This study has a number of limitations. Echocardiographic data were reported by the treating centers without independent core lab adjudication and follow-up echocardiographic data were missing in some cases.

The list of predictors of functional recovery after TAVI that has been investigated in the present analysis included valvular and cardiac function, as well as major comorbidities. However, markers of frailty and surrogates for arterial function and myocardial fibrosis were not included in our analysis. In addition, the socioeconomic and educational status of the patient as well as the involvement in regular exercise or rehabilitation programs might also play a role in determining the functional outcome of these patients. For future studies, we suggest to study the relation of those factors to the functional recovery after TAVI.

Clinicians assign a given patient to an NYHA class on the basis of their subjective interpretation of reported symptoms, and accordingly, interobserver variability of the functional assessment is a potential downside of this classification. In spite of this limitation, a higher NYHA class was shown in the present study to be a marker of objective adverse cardiac remodeling and, more importantly, of a higher mortality risk. It turns out that, in spite of its limitations, this simple tool that is still used to decide the timing of intervention (as recommended by clinical practice guidelines) can still be crucial in post-TAVI clinical assessment. Other more objective, more quantitative, and purely patient-reported multidimensional assessment tools have been suggested as better indices of the quality of life. These multidimensional tools (e.g., EuroQol-5L and SF-36 questionnaires) involve dimensions that are more determined by the extra-cardiac morbidities and general frailty (e.g., pain/discomfort, anxiety/depression, and independent self-care). TAVI that effectively relieves AS and its relevant symptoms and improves survival cannot reverse non-cardiac pathologies that profoundly impacts on the patient’s overall quality of life. Previous studies revealed that the general health status visual analog scale improves after TAVI by only 2.7–7.0% (mainly driven by improvements in mobility and usual activity dimensions, while the other dimensions showed only very modest change) [30] and that the EQ-5D index also shows a modest improvement (+7% at 1 year) [31].

## Conclusion

The majority of AS patients recover a normal functional status after TAVI despite the extensive comorbidities and the advanced cardiopathy they have at baseline. However, in a sub-group of patients, some degree of functional impairment persists and portends a diminished reverse cardiac remodeling and a lower survival. Chronic lung disease, anemia, atrial fibrillation, and a low-flow–low-gradient AS are baseline characteristics of this group of patients.

## Electronic supplementary material

Below is the link to the electronic supplementary material.

Supplementary Figure 1 (TIFF 163 kb)

Supplementary Table 1 (DOCX 24 kb)
