# Supplementary figures and images for: Prevalence, predictors, and prognostic implications of residual impairment of functional capacity after transcatheter aortic valve implantation
Source: Clin Res Cardiol. 2017 Apr 25;106(9):752–9. doi: 10.1007/s00392-017-1119-9 (PMC5565654; doi:10.1007/s00392-017-1119-9)

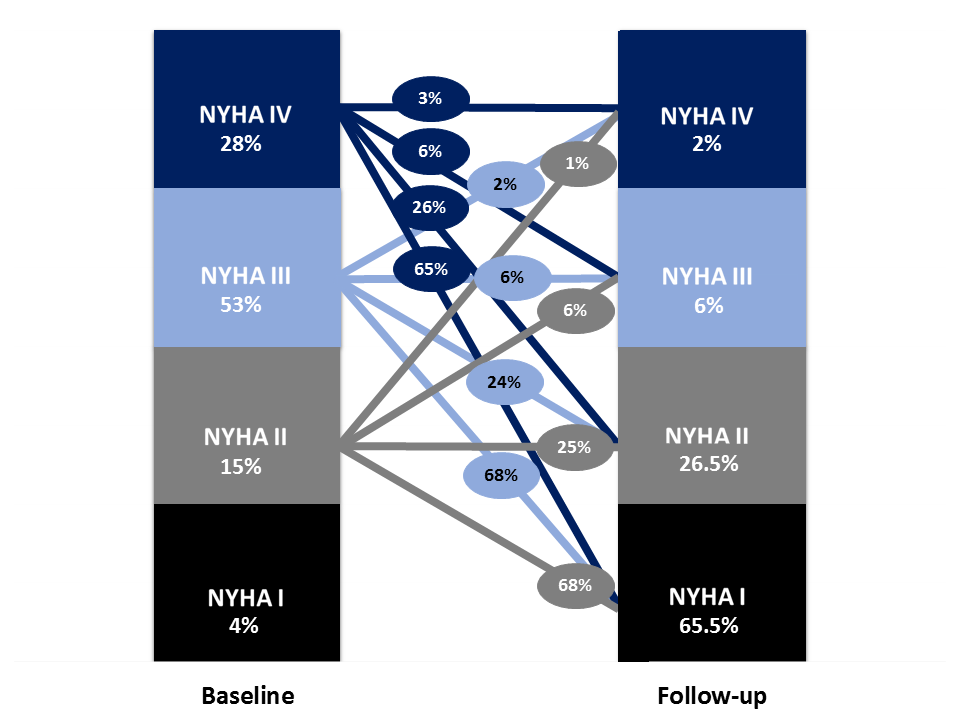

Supplement: Supplementary file 1 — Supplementary Figure 1 (TIFF 163 kb) [file 392_2017_1119_MOESM1_ESM.tif]
