# Supplementary material for: Prevalence, predictors, and prognostic implications of residual impairment of functional capacity after transcatheter aortic valve implantation
Source: Clin Res Cardiol. 2017 Apr 25;106(9):752–9. doi: 10.1007/s00392-017-1119-9 (PMC5565654; doi:10.1007/s00392-017-1119-9)
Supplement: Supplementary file 2 — Supplementary Table 1 (DOCX 24 kb) [file 392_2017_1119_MOESM2_ESM.docx]

**Supplementary Table.** Patient characteristics (baseline, periprocedural and follow-up) of the patients stratified according to the functional status at follow-up:

|  | NYHA I at latest follow-up (n=460) | NYHA II-IV at latest follow-up (n=242) | P |
| --- | --- | --- | --- |
| **Baseline characteristics:** | | | |
| Age at procedure | 81.1±7.6 | 81.7±6.8 | 0.343 |
| Male gender | 232(50%) | 120(50%) | 0.874 |
| Body mass index (kg/m^2^) | 26.5(4.7) | 25.8(4.6) | 0.064 |
| EuroSCORE | 14.5(8.9-25.5) | 17.9(11.1-29.3) | 0.001 |
| STS-PROM score | 7.0(4.0-14.9) | 7.1(4.4-14.3) | 0.694 |
| NYHA class |  | | 0.104 |
| *II* | 72(16%) | 34(14%) |  |
| *III* | 255(55%) | 122(50%) |  |
| *IV* | 114(25%) | 80(33%) |  |
| Coronary artery disease | 270(59%) | 141(58%) | 0.936 |
| Carotid artery disease | 65(14%) | 41(17%) | 0.321 |
| Peripheral arterial disease | 75(16%) | 39(16%) | 1.00 |
| Chronic obstructive pulmonary disease | 68(15%) | 56(23%) | 0.007 |
| Diabetes mellitus | 153(33%) | 66(27%) | 0.123 |
| Hypertension | 337(73%) | 185(76%) | 0.413 |
| Chronic kidney disease | 347(75%) | 190(79%) | 0.400 |
| Coronary artery bypass grafting | 92(20%) | 43(18%) | 0.546 |
| Previous percutaneous coronary intervention | 157(34%) | 80(33%) | 0.801 |
| Previous aortic valvuloplasty | 22(5%) | 17(7%) | 0.228 |
| Previous aortic valve replacement | 16(3%) | 12(5%) | 0.417 |
| Ejection fraction (%) | 59.4±14.6 | 57.3±15.5 | 0.072 |
| Ejection fraction<50% | 102(22.5%) | 63(26.6%) | 0.260 |
| LV mass index (g/m^2^) | 141.2±40.9 | 143.6±48.7 | 0.542 |
| Aortic valve area (cm^2^) | 0.66±0.18 | 0.67±0.21 | 0.398 |
| Transaortic valve mean PG (mmHg) | 50.3±15.6 | 47.6±16.6 | 0.034 |
| Low flow-low gradient AS* | 35(9%) | 37(18%) | 0.001 |
| Pulmonary hypertension | 89(19%) | 62(26%) | 0.066 |
| Moderate-severe aortic regurgitation | 56(13%) | 37(16%) | 0.242 |
| Moderate-severe mitral regurgitation | 80(18%) | 53(23%) | 0.154 |
| Cardiac rhythm |  | | 0.004 |
| *Sinus rhythm* | 376(82%) | 170(71%) |  |
| *Atrial fibrillation/flutter* | 46(10%) | 42(18%) |  |
| *Paced rhythm* | 36(8%) | 26(11%) |  |
| Hemoglobin (g %) | 11.9±1.8 | 11.6±1.8 | 0.009 |
| Creatinine Clearance (ml/min) | 50.4±23.1 | 45.7±18.8 | 0.004 |
| Brain natriuretic peptide**^¶^** (pg/ml) | 208(95-683) | 472(135-810) | 0.015 |
| **Periprocedural characteristics** | | | |
| Trans-femoral access | 434(94%) | 225(93%) | 0.509 |
| Transcatheter heart valve type |  | | 0.052 |
| *CoreValve* | 329(63%) | 193(37%) |  |
| *Sapien-XT* | 121(73%) | 44(27%) |  |
| Transcatheter heart valve diameter (mm) | 27.3±2.5 | 27.6±2.3 | 0.495 |
| Transcatheter heart valve oversizing (%) | 10.7(4.0-20.8) | 11.5(4.0-20.8) | 0.527 |
| Device success | 424(92%) | 211(87%) | 0.042 |
| Transaortic valve PG post-procedure^§^ | 0.0(0.0-6.0) | 3.0(0.0-8.0) | 0.005 |
| Hemoglobin pre-discharge (g %) | 9.8±1.7 | 9.5±1.5 | 0.022 |
| Creatinine pre-discharge (mg/dl) | 1.1(0.9-1.4) | 1.2(1.0-1.6) | 0.030 |
| Ejection fraction pre-discharge (%) | 61.6±12.9 | 59.2±14.4 | 0.041 |
| Moderate-severe aortic regurgitation pre-discharge | 23(5%) | 19(8%) | 0.132 |
| Moderate-severe mitral regurgitation pre-discharge | 62(15%) | 44(20%) | 0.094 |
| Medications prescribed at discharge |  | | |
| *ACEI/ARB* | 230(50.0%) | 118(48.8%) | 0.812 |
| *Beta blocker* | 143(31.1%) | 92(38.0%) | 0.077 |
| *Digitalis* | 14 (3.0%) | 15(6.2%) | 0.070 |
| *Warfarin* | 33(7.2%) | 27(11.2%) | 0.088 |
| *Diuretics* | 189(41.1%) | 124(51.2%) | 0.011 |
| **Follow-up** | | | |
| Days to last echocardiographic follow-up | 367(163-734) | 357(114-741) | 0.857 |
| Ejection fraction (%) | 62.1±12.6 | 57.9±15.6 | 0.002 |
| Ejection fraction<50% | 52(16) | 47(26) | 0.007 |
| LV diastolic diameter (mm) | 50.5±8.9 | 52.8±9.7 | 0.010 |
| Tran-aortic mean PG (mmHg) | 9.0(6.0-12.0) | 8.0(6.0-11.0) | 0.604 |
| LV mass index (g/m^2^) | 129.1±40.3 | 133.1±42.1 | 0.331 |
| Moderate-severe aortic regurgitation | 29(8%) | 22(12%) | 0.215 |
| Moderate-severe mitral regurgitation | 45(13%) | 47(26%) | <0.001 |
| All-cause death | 58(13%) | 79(33%) | <0.001 |
| Cardiac death | 23(5%) | 35(15%) | <0.001 |

Data presented as mean±SD, median(IQR), or n(%).

*Defined as a valve area ≤1.0 cm^2^ with a mean transvalvular pressure gradient ≤40 mmHg and an ejection fraction <50%.

**^§^**Invasively-measured peak pressure gradient.

**^¶^**Data available in 232 patients (158 in NYHA I group and 74 in NYHA II-IV group).

**Abbreviations:** ACEI, angiotensin converting enzyme inhibitor; ARB, angiotensin receptor blocker; AS, aortic stenosis; LV, left ventricle; NYHA, New York Heart Association; PROM, predicted risk of mortality; PG, pressure gradient; STS, Society of Thoracic Surgeons.
